# The value of social networks for men: concurrent and prospective associations with psychological wellbeing

**DOI:** 10.1186/s40359-025-02467-9

**Published:** 2025-02-20

**Authors:** Kayla Mansour, Christopher J Greenwood, Lauren M Francis, Gessica Misuraca, Khya Marabel-Whitburn, Craig A Olsson, Jacqui A Macdonald

**Affiliations:** 1 Deakin University, Deakin University, SEED Lifespan Strategic Research Centre, School of Psychology, Faculty of Health, Melbourne, Australia; 2 Centre for Adolescent Health, Murdoch Children’s Research Institute, Parkville, Australia; 3 Department of Paediatrics, Faculty of Medicine, Dentistry and Health Sciences, The Royal Children’s Hospital Campus, The University of Melbourne, Victoria, Australia; 4 Centre for Social and Early Emotional Development, School of Psychology, Faculty of Health, Deakin University, 221 Burwood Highway, Burwood, 3125 Australia

**Keywords:** Men, Longitudinal, Peers, Psychological wellbeing, Social connection

## Abstract

**Background:**

There is increased attention on psychological wellbeing as a public health priority. Here, we examine concurrent and prospective associations between social network connections and psychological wellbeing in a community sample of Australian men (*n* = 528).

**Methods:**

In regressions, using generalised estimating equations (GEEs), we assessed associations between men’s social network connections and psychological wellbeing across five annual timepoints. Social network connections were indicated by time spent with friends, close and extended network size, and activities with friends (physical activity, drinking alcohol, helping with a task, and sharing a meal). Psychological wellbeing was indicated by Ryff’s scales of environmental mastery and purpose in life.

**Results:**

Modelled concurrently, and longitudinally after adjusting for prior psychological wellbeing, time spent with friends and size of close and extended networks were positively associated with environmental mastery and purpose in life. In concurrent analyses, all activities except drinking with friends were positively associated with psychological wellbeing. Activities were not predictive longitudinally. Results did not differ by fatherhood or relationship status.

**Conclusions:**

Given concurrent and future effects of social network connections on men’s wellbeing, public health investment in opportunities that improve men’s social connectedness are recommended.

**Supplementary Information:**

The online version contains supplementary material available at 10.1186/s40359-025-02467-9.

Psychological wellbeing is increasingly recognised as a public health priority with governments in 12 countries now routinely undertaking recurrent wellbeing surveys (Trudel-Fitzgerald et al. 2019). Distinct from the absence of mental illness, psychological wellbeing emphasizes the presence of positive experiences that contribute to domain-specific (e.g., social, economic) and overall quality of life (Huppert 2009; Ryff 2013). Research to date links psychological wellbeing with better physical health (Friedman and Ryff 2012), reduced mortality (Friedman and Ryff 2012), reduced workplace stress (Piao and Managi 2022), higher labour productivity (Isham et al. 2020), and stronger personal relations (Lawton et al. 2017). Prospective studies point to the importance of social connectedness as a key factor in fostering development of psychological wellbeing (Lee and Martin 2023; Shankar et al. 2015; Stavrova and Luhmann 2016), yet longitudinal evidence specific to men is limited.

Social connections can be understood through three separate components suggested within Holt-Lunstad’s framework: structural components, such as time spent with others or the number of friends; functional components, which refer to the extent to which connections meet emotional needs; and relationship quality, encompassing factors like cohesion, conflict, and closeness (Hill and Dunbar 2003; Holt-Lunstad 2022). While these constructs are related, they remain distinct. Here, we examine the structural components of social connection among men. On average, men report smaller social networks and fewer interactions with peers when compared to women (McKenzie et al. 2018; YouGov 2019). Men also report a decline in the number of friends beginning in emerging adulthood (YouGov 2019). In a representative study in the United States (*N* = 1,254), 16% of men reported having no friends (YouGov 2019). Further, those aged between 20 and 37 years more commonly reported having no friends (22%) compared to those in both younger (16%) and older age groups (9–16%) (YouGov 2019). To date, longitudinal research focusing on structural indicators of men’s social networks (e.g., size, closeness, time spent) has primarily adopted a deficit-based approach and established that smaller social networks are linked to subsequent mental health problems (Mansour et al. 2023), and poorer quality of romantic relationships (Marabel-Whitburn, 2023). Less is known about the potential benefits that adequate social networks may confer on men’s psychological wellbeing, particularly in terms of two key indicators of wellbeing, environmental mastery and purpose in life. Such knowledge may inform and motivate investment in programs designed to reduce gender disparities in social connectedness.

To date, in mixed gender samples from across the lifespan, prospective evidence from cohort studies links social connectedness with later psychological wellbeing. For example, in adolescence, peer relationships have been associated with subsequent psychological wellbeing (Alsarrani et al. 2022). In older adulthood, social participation across the lifespan has been found to mitigate age-related declines in psychological wellbeing (Ang 2018). Further, a nationally representative sample of older US adults (over 50 years of age) found that social isolation and loneliness were longitudinally predictive of lower psychological wellbeing in later life (Hong et al. 2023). In these studies, two gaps are apparent; the first is that in contrast to contemporary recommendations (Haupt et al. 2024), even where gender gaps were examined, results have not been reported separately for male and female participants. The second gap is a lack of attention to the life stage known as established adulthood (age 30–45 years) (Mehta et al. 2020), identified as a developmental ‘crunch time’ for relationship and career consolidation and typically a transition to parenthood (Mehta et al. 2020). At this life stage, the social and economic benefits of psychological wellbeing, including environmental mastery and purpose in life, for men, their families and their community, may be particularly heightened. Further, given typical shifts in social investment from peers to family when men move into stable relationships or become fathers (McKenzie et al. 2018), it may be particularly beneficial to examine the varying degrees of influence social connections with friends have on men’s psychological wellbeing when they experience these milestones. Here, we examine the relationships between men’s social connections and environmental mastery and purpose in life, two core constructs of psychological wellbeing.

Environmental mastery refers to a person’s sense of control and competence in managing their environment (Ryff 2013). High environmental mastery is characterised by feeling capable of handling life’s challenges, adapting to new situations, and making choices that positively impact one’s own life (Ryff 2013). A study of 500 adults found those who experienced positive relations with others reported increased environmental mastery over time (Garcia et al. 2014). Structural indicators of social networks have also been linked to environmental mastery (Ashida et al. 2019). In a United States representative sample (*N =* 6,928) a stronger sense of social isolation (characterised by lower numbers of close friends, close relatives, individuals seen at least once during the last month) was found to lead to a decrease in perceived mastery over the environment (Kaplan et al. 2008). Further, having larger extended networks of members who provided companionship has been associated with higher environmental mastery in older adults (Ashida et al. 2019). One explanation may be benefits that arise from ties beyond immediate close connections that serve to expand access to resources and information (Granovetter 1973), important foundations in the development of environmental mastery. Across these studies, results were only presented for the full mixed gender samples leaving a gap in understanding these associations for men. Such connections may be particularly beneficial for men at the life stage of ‘established adulthood’ where family and workplace developmental milestones commonly require transitions through psychological uncertainty and rapid skill acquisition (Kings et al. 2017).

Purpose in life contributes to overall wellbeing by providing individuals with a sense of direction, a source of motivation, and a framework for making decisions that align with their values and long-term aspirations (Ryff 1995, 2013). Examining these dynamics among men in established adulthood may be particularly important given the unique social and emotional challenges encountered during this life stage (Mehta et al. 2020 Purpose in life is a fundamental construct within conceptualisations of a meaningful and fulfilling life and a key component of psychological wellbeing (Baumeister, 2002; Lambert et al. 2013). Social connections provide a vital support system that may nurture and reinforce one’s sense of purpose. Stavrova and Luhmann (2016) theorise that collective connectedness (i.e., belonging to a network of multiple friendships) increases the likelihood of identification with a group and reduces the potential for meaninglessness in one’s existence. They found, in a study of 4,963 adults, that those who reported higher levels of social connection at one point in time were more likely to experience an increased meaning in life over the course of several years (Stavrova and Luhmann 2016). On the reverse, socially isolated older adults (*n* = 43) qualitatively reported experiencing less purpose in life (Machielse 2018). By examining how social connections are linked to purpose in life, we may better understand the pathways through which men’s social relationships may contribute to their psychological wellbeing.

Men’s sense of environmental mastery and purpose in life may be further linked to the activities they engage in when they spend time with those in their social networks. Research indicates that men tend to prioritize instrumental or tangible means of connecting with peers and often base their time spent with friends on shared interests or activities (Arbes et al. 2014). A United Kingdom study of 2000 adults found that eating more meals with other people had positive effects on satisfaction with life, happiness, sense of engagement with and trust of one’s local community (Dunbar 2017). Engaging in physical activity with friends has benefits for physical and psychological health but may have particular benefits for men’s mastery and purpose in life given physical pursuits are often accompanied by goal setting, skill development and collective accomplishments. Despite this, less is known about which types of social activities that may be associated with men’s environmental mastery and purpose in life in the period proximal to the activity and over time.

In this study, we use longitudinal data from an Australian cohort of adult men that spanned across five yearly assessments to investigate cross-sectional and prospective associations between men’s social network connections and their psychological wellbeing.

In cross-sectional and longitudinal analyses, we predict the following:

- H1: Time spent with friends will be positively associated with men’s psychological wellbeing (i.e., environmental mastery and purpose in life).
- H2: Close and extended friendship network size will be positively associated with men’s psychological wellbeing.
- H3: Engagement in various types of activities with friends will be positively associated with men’s psychological wellbeing.

We further explored whether these hypothesised associations (H1, H2, H3) differed by fatherhood or relationship status.

## Method

### Participants

Participants were from the Men and Parenting Pathways (MAPP) Study (*N* = 608), a five-year longitudinal cohort study that examined the mental health and wellbeing of Australian men across the peak age for transitioning to fatherhood (33 years) (Australian Bureau of Statistics 2019; Macdonald, 2021). MAPP eligibility required participants to be aged between 28 and 32 years at commencement, male, and English- speaking, and an Australian resident. These Australian men were invited to participate regardless of parenting status or intention to have children/more children. The MAPP study used recruitment strategies previously reported by the Australian Longitudinal study on Women’s health (Loxton et al. 2015) including promotion through partnership organisations, and both traditional and social media (Macdonald, 2021). The resulting sample was representative of key demographics, but overrepresented Australian born men and men in paid employment (Macdonald, 2021). To be included in the current study, participants were required to have data on at least one indicator of social connection at any one of the five waves and at least one psychological wellbeing outcome in one of the five waves. This resulted in an analytic sample of 528 participants. Sample characteristics can be found in Table 1. In comparison to the non-analytic sample (*n* = 80), the analytic sample had slightly higher household income and education (*p* < .001), were older (*p* = .046), and fathers had younger children (*p* = .002).

**Table 1:** Demographic characteristics of the full MAPP study sample and analytic sample at baseline (Wave 1)

	Full MAPP Sample (*N* = 608)		Analytic Sample (*n* = 528)	
Demographic Variable	*n*	%	*n*	%
Birthplace (Australia)	536	88.16	470	89.02
Sexual orientation (Heterosexual/straight)	540	91.84	472	89.39
Relationship status (In a relationship)	483	79.83	424	80.30
Fatherhood Status (Father)	241	39.64	216	40.91
Education (> Year 12)	469	77.14	421	79.73
Household income ($AUD60,000+)	446	73.36	405	76.70
	*n*	*M* (*SD*)	*n*	*M* (*SD*)
Age of participant, years	608	29.85 (1.32)	528	29.89 (1.32)
Age of youngest child, years	234	2.53 (2.51)	217	2.39 (2.32)

Statistics reported in this table are based on non-imputed data (i.e., available case)

### Measures

#### Outcome measure

Psychological wellbeing was assessed across the five waves using two subscales from Ryff’s Psychological Wellbeing Scale (Ryff 1995), Environmental Mastery and Purpose in Life. The sub-scale of Environment Mastery assesses the belief of one’s ability to manage life events and contains six items, such as *“In general*,* I feel I am in charge of the situation in which I live”*. Purpose in Life assesses one’s sense of purpose and meaningfulness in life and contains five items, such as, *“I enjoy making plans for the future and working to make them a reality”.* Responses across both subscales range from 1 = *Strongly Disagree* to 7 = *Strongly Agree*, with higher scores reflecting a greater sense of environmental mastery and purpose in life. Across the five waves of data, both the mastery and purpose in life subscales showed good internal consistency (α range = 0.83 to 0.86 and 0.81 to 0.83, respectively).

#### Predictor measures

##### Time spent with friends

At each wave, participants were asked to report on their time spent with friends, answering the question: “How many hours a week on average, outside of work, would you spend in the company of friends?”. An open field response option was used and as such some participants reported times that exceeded a logical range. To address this, data were winsorised at a point derived by averaging the value at three standard deviations from the mean across the five timepoints (Dixon and Yuen 1974). This resulted in an upper limit of 30 h, which is more than twice the average number of hours per week (13.29 h) that men of a similar age (25 to 34 years) reported spending with friends in the Australian National Time Use Survey (Australian Bureau of Statistics 2008).

##### Close friendship network size

At each wave, participants were asked to record their close friendship network size with the question: “How many people would you count as close friends?”. Responses were given across six options (“*None”*, “*1 or 2”*,* “3 to 5”*,* “6 to 10”*,* “11 to 15”*, and *“16 or more”).* As per prior studies (Mansour et al. 2023; Marabel-Whitburn, 2023), we coded these options as the midpoint of the range of each response option to provide a meaningful average score, i.e., 0 = “*None”*, 1.5 = “*1 or 2”*, 4 = *“3 to 5”*, 8 = *“6 to 10”*, and 13 = *“11 to 15”*,. For the final response category, for which no mid-point exists, the same midpoint interval used in the previous categories was applied (i.e., 18 = *“16 or more*”).

##### Extended friendship network size

Extended friendship network size was recorded at each wave using the question: “How many people would you count as friends but not so close?”. Responses were given across six options (“*None”*, “*1 or 2”*,* “3 to 5”*,* “6 to 10”*,* “11 to 15”*, and *“16 or more”).* For analysis we coded these options in line with the coding reported above for the Close Friendship Network Size variable.

##### Activities with friends

Activities with friends was reported at each wave using the question: “When you spend time with your friends, what do you usually do?” Participants were instructed to ‘select all that apply’ for the following activities: Play sport/do physical activity; Drink alcohol; Go out for meals; Help each other with a task. A final option of ‘if other, please specify’ was offered, and responses were recoded into the previous four categories where appropriate.

### Moderating variables

#### Fatherhood status

Participants reported their fatherhood status at each wave. Responses were dichotomised to 0 = *not a father*, 1 = *father (of a biological*,* step*,* or adoptive child)* and were included in analyses at the same wave of the predictor (i.e., social network variables).

##### Relationship status

Participants reported their relationship status at each wave. Responses were dichotomised to *0 = in a relationship status*,* 1 = not in a relationship*, and were included in analyses at the same wave of the predictor (i.e., social network variables).

#### Potential confounder variables

Prior research has identified links between key socio-demographic indicators of both social network connectedness and psychological wellbeing that may confound associations. We adjusted for these in regression analyses using baseline (i.e., Wave 1) assessments to ensure they were not on the causal pathway between predictor and outcome (VanderWeele 2019). These included annual household income (0 = greater than or equal to $AUD 60,000 per annum, 1 = less than $AUD 60,000 per annum), birthplace (0 = Australia, 1 = not Australia), and education (0 = greater than high school education, 1 = less than high school completion).

#### Statistical analysis

All analyses were conducted in Stata version 15.1. We estimated linear regression models using Generalised Estimating Equations (GEEs) with robust variance estimators and an exchangeable working correlation to account for within person clustering (i.e., multiple waves of data for each participant). Specifically, to examine cross-sectional associations, in separate models, environmental mastery and purpose in life were regressed onto each concurrently assessed social connection variable. Models were first conducted adjusting only for wave, and then for wave plus all potential confounders and the main effect of the moderators. Further, to examine if associations were also apparent longitudinally after controlling for earlier levels of the outcome, models were repeated but with psychological wellbeing regressed on to prior wave measurements of social connection (predictor) and psychological wellbeing (additional confounder). Tests of interactions between predictors and wave, predictors and fatherhood status, and predictors and relationship status were included in the models to examine whether the strength or direction of these associations varied. An alpha level of 0.05 was used for significance testing.

Missing data ranged from 0 to 31.44% in the full analytic sample, for which missing within participants of each wave remained low (i.e., (wave 1: 0–8.14%, wave 2: 0.21–6.78%, wave 3: 4.44–11.21%, wave 4: 2.22–7.90%, wave 5: 0.50–9.73%). To address missing data, multiple imputation was performed, with 20 complete datasets imputed (Rubin 2004). Due to the multiple waves of related measurements, we used a two-fold fully conditional specification algorithm (Welch et al. 2014). Sensitivity analyses were conducted to compare results using imputed and available case data. As per the MAPP data sharing policy, ethics approvals do not include participant consent for public availability of data, however, requests for reuse of data for validation, verification or confirmation of past research are supported (Macdonald, 2021).

## Results

### Descriptive statistics

A summary of outcome and predictor variables at each wave including means, standard deviations, and missing data can be found in Supplementary Table S1. On average, across the five waves, men spent approximately 4.9 h a week with friends outside of work. They also reported an average of 4 close friends and 10 extended friends. Results did not differ between imputed data and available case data, as such only imputed results are reported. See Supplementary Material for available case results.

### Social networks and environmental mastery

Table 2 presents confounder unadjusted and adjusted GEE model estimates, in which environmental mastery was regressed in cross-sectional and longitudinal analyses onto social network variables. In tests of interactions (predictor by wave), none of the associations differed by wave, fatherhood status or relationship status (*p* > .05), therefore only main effects are presented. In adjusted cross-sectional analyses, there were significant positive associations between each of the social network variables (*b* range = 0.12 to 1.3), except for drinking with friends. In adjusted longitudinal analyses, positive associations remained for time spent with friends, close friendship network size, and extended friendship network size (*b* range = 0.06 to 0.18). Evidence did not support longitudinal effects for any of the types of activities with friends.

**Table 2:** Linear GEE Model of Social Network Variables Predicting Environmental Mastery across five waves (*n* = 528)

Variable	Cross-sectional						Longitudinal^a^					
	Confounder unadjusted			Confounder adjusted			Confounder unadjusted			Confounder adjusted		
	*b*	95% CI	*p*	*b*	95% CI	*p*	*b*	95% CI	*p*	*b*	95% CI	*p*
**Level of Investment**												
** Time Spent**	0.13	0.08, 0.18	**< 0.001**	0.06	0.02, 0.10	**0.007**	0.05	0.01, 0.09	**0.013**	0.06	0.02, 0.10	**0.007**
** Close Network Size**	0.39	0.22, 0.46	**< 0.001**	0.18	0.08, 0.28	**0.001**	0.16	0.07, 0.25	**< 0.001**	0.18	0.08, 0.28	**0.001**
** Extended Network Size**	0.12	0.06, 0.19	**< 0.001**	0.07	0.02, 0.12	**0.007**	0.07	0.03, 0.11	**0.001**	0.07	0.02, 0.12	**0.007**
**Type of Investment**												
** Physical Activity**	1.43	0.85, 2.01	**< 0.001**	1.3	0.72, 1.88	**< 0.001**	0.39	−0.13, 0.91	0.143	0.29	−0.23, 0.83	0.273
** Drink Alcohol**	0.38	−0.16, 0.92	0.165	0.36	−0.18, 0.90	0.194	0.32	−0.19, 0.82	0.218	0.27	−0.23, 0.78	0.286
** Go for a Meal**	1.38	0.87, 1.90	**< 0.001**	1.3	0.78, 1.82	**< 0.001**	0.21	−0.32, 0.76	0.432	0.13	−0.44, 0.71	0.644
** Help with a Task**	0.76	0.23, 1.29	**0.005**	0.76	0.23, 1.29	**0.005**	0.16	−0.43, 0.74	0.596	0.15	−0.44, 0.74	0.612

All analyses adjusted for wave. Confounder adjusted analyses additionally adjusted for birthplace; education; income. Analyses are based on imputed data

^a^In longitudinal analyses lagged predictors were assessed waves 1–4, one wave prior to outcomes and included additional adjustment for prior reports of environmental mastery

### Social networks and purpose in life

Table 3 presents confounder unadjusted and adjusted GEE model estimates, in which purpose of life as regressed in cross-sectional and longitudinal analyses onto social network variables. In tests of interactions (predictor by wave), none of the associations differed by wave, fatherhood status or relationship status (*p* > .05), therefore only main effects are presented. In adjusted cross-sectional analyses, there were significant positive associations between each of the social network variables (*b* range = 0.03 to 0.68), except for extended friendship network size and drinking with friends. In adjusted longitudinal analyses, positive associations were apparent for time spent with friends, close friendship network size, and extended friendship network size (*b* range = 0.04 to 0.11), although it is noted that the evidence for time spent with friends was weaker (*p*^unadj^ = 0.059; *p*^adj^ = 0.057). Evidence did not support longitudinal effects for any of the types of activities with friends.

**Table 3:** Linear GEE Model of Social Network Variables Predicting Purpose in life across 5 waves (*n* = 528)

Variable	Cross-sectional						Longitudinal^a^					
	Confounder unadjusted			Confounder adjusted			Confounder unadjusted			Confounder adjusted		
	*b*	95% CI	*p*	*b*	95% CI	*p*	*b*	95% CI	*p*	*b*	95% CI	*p*
**Level of Investment**												
** Time Spent**	0.07	0.03, 0.11	**0.002**	0.07	0.03, 0.12	**< 0.001**	0.04	0.00, 0.08	**0.059**	0.04	0.00, 0.09	**0.057**
** Close Network Size**	0.2	0.10, 0.30	**< 0.001**	0.19	0.11, 0.28	**< 0.001**	0.16	0.07, 0.25	**< 0.001**	0.11	0.03, 0.18	**0.007**
** Extended Network Size**	0.04	−0.12, 0.09	0.176	0.03	−0.02, 0.08	0.202	0.06	0.02, 0.09	**0.003**	0.05	0.01, 0.08	**0.006**
**Type of Investment**												
** Physical Activity**	0.78	0.30, 1.25	**0.001**	0.68	0.21, 1.14	**0.004**	0.28	−0.14, 0.72	0.192	0.22	−0.22, 0.66	0.324
** Drink Alcohol**	−0.02	−0.49, 0.45	0.936	−0.01	−0.48, 0.45	0.955	0.29	−0.13, 0.71	0.178	0.29	−0.13, 0.71	0.175
** Go for a Meal**	0.9	0.44, 1.36	**< 0.001**	0.87	0.40, 1.33	**< 0.001**	0.21	−0.22, 0.64	0.341	0.15	−0.31, 0.61	0.524
** Help with a Task**	0.68	0.22, 1.13	**0.004**	0.69	0.25, 1.14	**0.002**	0.04	−0.42, 0.50	0.860	0.04	−0.42, 0.49	0.875

All analyses adjusted for wave. Confounder adjusted analyses additionally adjusted for birthplace; education; income. Analyses are based on imputed data

^a^In longitudinal analyses lagged predictors were assessed waves 1–4, one wave prior to outcomes and included additional adjustment for prior reports of purpose in life

## Discussion

The current study addresses a gap in knowledge about the importance of men’s social network connections to their psychological wellbeing. Broadly, we found that time spent with friends and the number of friends in close and extended social networks were associated with both environmental mastery and purpose in life concurrently and one year later. We also found that sharing a meal and engaging in physical activity with friends, as well as helping a friend were each concurrently, but not prospectively associated with psychological wellbeing. These findings were evident regardless of fatherhood or relationship status, supporting the proposition that interventions that build men’s social connections, may not only reduce mental health burden but may also improve psychological wellbeing.

### Time spent with friends

A key finding of this study is that men’s time spent with friends is concurrently and longitudinally linked to both environmental mastery and purpose in life. Across the five timepoints, men spent an average of 4.9 h with friends a week. Our findings extend on conclusions in a recent review of 38 studies that found time spent with friends was associated with subjective wellbeing in the moment and 6 and 12 months later, specifically life satisfaction and positive affect (Pezirkianidis et al. 2023). Building and maintaining friendships requires effort and investment (Lu et al. 2020). Investing in time spent with friends involves nurturing and enriching relationships that are important. For men, spending time with friends is often instrumental or centred around goal-orientated engagement (Arbes et al. 2014). In this sense, time with friends may contribute to a sense of purpose and mastery by fostering shared experiences, collaborative achievements, and a sense of belonging to something larger than oneself (Lambert et al. 2013). Our findings suggest that this may provide a framework through which men are able to find meaning and purpose in their lives and feel a sense of control both in the moment and overtime. In addition, when interacting with peers, men may experience an opportunity for social learning whereby observing others’ behaviours, skills, and strategies, acquisition of new knowledge and abilities may be achieved (Anderson and Fowers 2020; Durrah 2023). Here, observational learning may enhance skills and understanding, fostering a sense of mastery and purpose. This is in line with previous research that demonstrates a direct association between observational learning and self-efficacy (Kwon et al. 2022), a core component of mastery and purpose in life (Bandura 1977; DeWitz et al. 2009; Sabouripour et al. 2021).

### Close friendship networks

Larger networks of close friends were also associated with greater environmental mastery and purpose in life in the moment and a year later. For context, in our study at wave 1, two thirds of men reported between 3 and 10 close friends. Parallel to the current finding, in longitudinal research, low numbers of close friends reported by men have been shown to predict subsequent depressive symptoms (Mansour et al. 2023). Our findings extend on the prior mental health focus of research and demonstrates the importance of close friendship networks for positive outcomes. We provide insight into possible opportunities to promote flourishing through men’s social connections which may have extended implications in various related aspects of men’s lives including their physical health, family life and the workplace. Our findings may be explained by prior longitudinal research (*n* = 169) that showed having a close circle of friends was predictive of self-worth in adulthood (Narr et al. 2019). Feelings of self-worth and confidence may translate into a greater sense of mastery as one may feel more capable of handling various life situations and stressors (Morales-Rodríguez et al. 2020). In addition, close friendships often involve a level of accountability (Kelly 2023). A qualitative paper examining the importance of adult friendships found a key benefit of close friends is having a sense of accountability whereby ‘hard truths’ can be expressed (Kelly 2023). Having an increased number of close ties may therefore provide encouragement to set and achieve personal goals through accountability, whether related to work, education, or personal development, in turn, fostering not only a sense of control over one’s life but also a heightened level of purpose in life.

### Extended friendship networks

When we examined men’s extended social networks, we found that larger networks were linked to higher levels of environmental mastery in the moment and a year later. Men who are embedded within diverse and expansive social networks tend to have access to a wider range of experiences, information, and opportunities (Granovetter 1973; Sandstrom and Dunn 2014). This exposure may contribute to a sense of control over the environment by increasing knowledge, enhancing problem-solving skills, and fostering adaptability. Furthermore, research suggests that when there is reduced emphasis on forming strong social bonds (as per with weaker ties), individuals may be more likely to participate in discussions centred around important subjects (Small 2013). Consequently, this may have a significant impact on facilitating access to valuable information and support and in turn have a long-term impact on men’s purpose in life.

In relation to purpose in life, the association with extended friendship networks was only apparent longitudinally. It is important to note that the effect size was small, however there may be some benefit over time for extended friendship groups in fostering purpose in life. Prior research that found when examining various network ties including collective, relational, and intimate, only collective connectedness (defined by community interaction) was predictive of meaning in life (Stavrova and Luhmann 2016). Interacting with a larger social circle provides exposure to a wider range of perspectives and experiences which may broaden one’s understanding of the world and promote meaning in various environments and situations.(Sandstrom and Dunn 2014). It may be that this process takes time to develop and therefore may explain why this relationship is only predictive longitudinally. This pattern aligns with the concept of developmental cascades, which are well-documented in child and adolescent research, where positive social connections contribute to positive developmental outcomes over time (Blair et al. 2015). A similar cascading process may occur in adulthood, suggesting that extended social networks in men may initiate a series of positive adult developmental outcomes that enhance purpose in life over time.

### Activities with friends

Of the activities that we explored that men engage in with friends, physical activity had the strongest relationship with concurrent psychological wellbeing (both mastery and purpose in life). A previous study of 489 young adults found that men’s physical activity was positively associated with their psychological wellbeing, including mastery and purpose in life (Granero-Jiménez et al. 2022). These findings suggest that physical activity alone may be beneficial in itself for men’s psychological wellbeing due to its positive benefits including endorphin release (Rebar et al. 2015), increased brain functioning (Valkenborghs et al. 2019) and greater self-efficacy (Difrancesco et al. 2022). Furthermore, research found that men are more likely to engage in physical activity due to intrinsic motivations, such as improving one’s image or personal growth, in comparison to women (Granero-Jiménez et al. 2022). By engaging in physical activity with friends, these motivations may increase feelings of mastery and purpose in life through competition, achieving personal bests and goal-orientation (Granero-Jiménez et al. 2022).

Sharing a meal with friends (commensality) was also important for men’s psychological wellbeing in the moment. Men who reported sharing meals with friends experienced greater environmental mastery and purpose in life. Past research has consistently shown a link between commensality and life satisfaction – a facet of wellbeing (Dunbar 2017; Kim 2020). Furthermore, commensality often involves sharing food, which is an act of generosity and altruism (Jönsson et al. 2021). Engaging in this act can contribute to a sense of purpose by promoting a feeling of contribution and making a positive impact on others. Sharing cultural or religious rituals and traditions may also occur when having meals with friends (Jönsson et al. 2021). These shared values and beliefs may contribute to a sense of purpose by providing individuals with a connection to something larger than themselves.

Helping a friend with a task was also found to be beneficial for men’s psychological wellbeing at the time. In a past study, helping a friend with a task was found to be protective against depressive symptoms (Mansour et al. 2023). We extend on these findings, linking the provision of social support to greater feelings of mastery and purpose in life. For men, successfully assisting a friend and contributing to their success may provide a sense of achievement and accomplishment (Cai and Lian 2022). This experience may provide a sense of motivation to continue learning and improving their mastery (Curry et al. 2018). Further, it may also provide a sense of purpose through feeling needed and valued in their friendships. This is in line with a review of 21 studies that found a link between volunteering and quality of life, 3 of which were focused specifically on psychological wellbeing (Cattan et al. 2011).

Our study found that drinking alcohol with friends had no relationship with environmental mastery and purpose in life. The impact of alcohol consumption with friends on psychological wellbeing may depend on the frequency and quantity of consumption, as well as the context in which it occurs (Dunbar et al. 2017). Further, some individuals may derive a sense of enjoyment or relaxation from drinking with friends, while others may not (Dunbar et al. 2017). Given these facets were not explored in this study, the varying subjective experiences of individuals in response to alcohol consumption may contribute to the lack of association with psychological wellbeing.

### Fatherhood and relationship status

It is important to note that none of our study results differed depending on relationship status and fatherhood status. Fatherhood and dyadic relations are two normative experiences during the life stage of ‘established adulthood’ that may reduce the capacity for social network investment (Mehta et al. 2020). The findings of this study suggest that the implications of men’s social network connections on their psychological wellbeing may be apparent for all men regardless of whether they enter these milestones or not. This is in line with theory that posits social connectedness is an essential and fundamental human need (Baumeister and Leary 1995). Our findings also build on prior deficit approaches in research that too demonstrated the relationship between poor social network connections and mental health problems in men who were and were not fathers and those with and without romantic partners (Mansour et al. 2023). In this study, we see the presence of these social networks promoting psychological wellbeing, which may in turn, foster a sense of flourishing.

### Implications

Commonly, studies of men take a deficit model approach (e.g., focusing on substance misuse, mental illness, absent fathering) (Francis et al. 2023; Oliffe et al. 2019; Rowland et al. 2023); however, it is important to acknowledge and explore how men thrive, how they contribute to society and opportunities to promote wellbeing. Our key findings demonstrate the importance of examining social connections for men’s psychological wellbeing. This aligns with strength-based models of care whereby recognizing and building upon men’s inherent strengths, capabilities, and positive attributes allows for the possible promotion of their mental health, overall wellbeing, and resilience by utilising their existing resources and capacities (Misuraca et al. 2024; Seidler et al. 2018; Wilson et al. 2022). However, poorer connections may originate from socialized masculine norms, where mutual reliance and emotional disclosure with peers are less common (Gillespie et al. 2015). These norms may reduce opportunities for men to form and sustain meaningful social connections, which could negatively impact their psychological wellbeing. Considering the notable successes seen in social programs for younger males in schools (Gwyther et al. 2019), and for older Australian men through programs such as Men’s Shed (Kelly et al. 2021), there may be considerable advantages in creating social initiatives tailored for men in the stage of ‘established adulthood,’ where time constraints are typically more pronounced. Moreover, in this phase of life, a deepening commitment to partners, children, and/or career is often experienced (Mehta et al. 2020). Promoting psychological wellbeing may have far-reaching positive impacts into communities and societies, with numerous benefits across the workplace and family life. For example, investment in social networks has also been linked to improved romantic relationship quality (Marabel-Whitburn, 2023). We examined these men with a focus on their individual contribution to their own wellbeing; however, future research may benefit from focusing on understanding the systems and combined contribution to wider wellbeing (e.g., family wellbeing). Furthermore, our research suggests value in including social connectedness as an indicator in wellbeing surveys. Future research may also benefit from examining additional components of social connection including the functions or the quality of connection and how these are linked to men’s psychological wellbeing.

### Strengths and limitations

The use of rare prospective data in an adult sample of men is a key strength of this study. Further, by examining the associations between men’s social network connections and psychological wellbeing, while controlling for prior reports of psychological wellbeing, along with a range of potential confounding variables, we have been able to account for reverse causation. To our knowledge, this is the first study to do this while examining the relationship between these variables. By doing so, we can have increased confidence in the likely causal links between the time spent with friends and the number of close and extended friend networks and subsequent psychological wellbeing. Despite this, there is still the potential for unmeasured confounders which is apparent in any observational research. Therefore, future research may benefit from examining the causal links between social networks and psychological wellbeing with a focus on a different range of confounders or using alternative approaches. Further, while there was some loss to follow-up, cohort studies with male participants tend to have lower retention rates compared to the current study (Teague et al. 2018). Due to the longitudinal nature of this research, missing data had the potential to introduce bias into estimates. To mitigate this, multiple imputation was performed which has been found to result in unbiased estimates (Bemaards et al. 2007) .

This study may also be limited by examining only two of the six subscales of the Ryff Scales of Psychological Wellbeing (Ryff 1995). Although there is considerable conceptual overlap and correlation between the subscales (Gao and McLellan 2018), the Ryff Scales aim to capture the multidimensional nature of psychological wellbeing, and each subscale is intended to measure a unique facet of this overarching construct (Ryff 2013). Despite this, environmental mastery and purpose in life were specifically chosen in this study due to the importance these facets have on men at this ‘crunch time’ period of established adulthood (Simard et al. 2022; Zeldin et al. 2008), but also to reduce respondent burden given the extensive range of domains examined in the study survey (Macdonald, 2021). Further in a study examining men and women’s self-attributions of so-called masculine and feminine traits, men’s (*n =* 1,700) identification with masculine traits was most strongly associated with environmental mastery and purpose in life out of Ryff’s six psychological wellbeing constructs (Matud et al. 2019).

## Conclusion

This study sheds light on the nuanced relationship between men’s social networks and their psychological wellbeing. Our findings underscore the importance of the size, time engagement and activity base of men’s social networks in promoting both immediate and long-term aspects of psychological wellbeing, specifically environmental mastery and purpose in life. Given that international evidence suggests men of this age have poor engagement in friendship networks (McKenzie et al. 2018; YouGov 2019), public health investments in avenues that improve social connectedness are required, particularly those with a focus on the structural indicators including the time spent and size of men’s social networks.

## Supplementary Information

Supplementary Material 1.

Supplementary Material 2.

## Data Availability

As per our data sharing policy, MAPP ethics approvals do not include participant consent for public availability of our data, however, requests for reuse of data for validation, verification or confirmation of past research are supported.
