# Supplementary material for: The value of social networks for men: concurrent and prospective associations with psychological wellbeing
Source: BMC Psychol. 2025 Feb 20;13:142. doi: 10.1186/s40359-025-02467-9 (PMC11843785; doi:10.1186/s40359-025-02467-9)
Supplement: Supplementary file 1 — Supplementary Material 1. [file 40359_2025_2467_MOESM1_ESM.docx]

| Supplementary Table 1. *Summary Statistics of Predictor and Outcome Variables* | | | | | |
| --- | --- | --- | --- | --- | --- |
| Variable |  | *M* (*SD*) | Range | Missing Data  Analytic Sample (%) | Missing Data Within Waves*  (%) |
| Mastery |  |  |  |  |  |
|  | Wave 1 | 28.18 (6.52) | 7 –42 | 0 | 0 |
|  | Wave 2 | 28.13 (6.22) | 7 –42 | 10.80 | 0.21 |
|  | Wave 3 | 27.9 (6.31) | 7 –42 | 19.70 | 0.93 |
|  | Wave 4 | 23.44 (6.13) | 7 –42 | 29.36 | 7.90 |
|  | Wave 5 | 27.46 (7.13) | 7 –42 | 28.22 | 5.49 |
| Purpose in Life | |  |  |  |  |
|  | Wave 1 | 29.38 (6.21) | 7 –42 | 8.14 | 8.14 |
|  | Wave 2 | 29.59 (6.22) | 7 –42 | 16.67 | 6.78 |
|  | Wave 3 | 29.41 (6.44) | 7 –42 | 28.03 | 11.21 |
|  | Wave 4 | 29.35 (6.30) | 7 –42 | 25.00 | 2.22 |
|  | Wave 5 | 28.97 (6.51) | 7 –42 | 24.43 | 0.50 |
| Time Spent with Friends | |  |  |  |  |
|  | Wave 1 | 6.06 (6.41) | 0 - 30 | 3.41 | 3.41 |
|  | Wave 2 | 4.88 (5.14) | 0 - 30 | 13.83 | 3.60 |
|  | Wave 3 | 4.74 (5.15) | 0 - 30 | 24.43 | 6.78 |
|  | Wave 4 | 4.75 (5.70) | 0 - 30 | 28.98 | 7.41 |
|  | Wave 5 | 4.09 (5.31) | 0 - 30 | 31.44 | 9.73 |
| Close Friendship Network Size | | |  |  |  |
|  | Wave 1 | 4.36 (3.14) | 0 - 18 | 1.70 | 1.70 |
|  | Wave 2 | 4.03 (2.75) | 0 - 18 | 11.93 | 1.48 |
|  | Wave 3 | 3.89 (2.90) | 0 - 18 | 22.54 | 4.44 |
|  | Wave 4 | 4.15 (3.18) | 0 - 18 | 27.65 | 5.68 |
|  | Wave 5 | 4.15 (3.20) | 0 - 18 | 29.17 | 6.73 |
| Extended Friendship Network Size | | |  |  |  |
|  | Wave 1 | 11.03 (5.81) | 0 - 18 | 1.70 | 1.70 |
|  | Wave 2 | 10.63 (5.81) | 0 - 18 | 11.74 | 1.27 |
|  | Wave 3 | 10.12 (5.75) | 0 - 18 | 22.73 | 4.67 |
|  | Wave 4 | 9.83 (5.86) | 0 - 18 | 27.65 | 5.68 |
|  | Wave 5 | 9.45 (5.84) | 0 - 18 | 29.17 | 6.73 |
|  |  | *n* | % |  |  |
| Physical Activity with Friends (Yes) | | |  |  |  |
|  | Wave 1 | 145 | 27.46 | 3.41 | 3.41 |
|  | Wave 2 | 155 | 34.07 | 13.83 | 3.60 |
|  | Wave 3 | 145 | 36.34 | 24.43 | 6.78 |
|  | Wave 4 | 138 | 36.80 | 28.98 | 7.41 |
|  | Wave 5 | 116 | 32.04 | 31.44 | 9.73 |
| Drinking Alcohol with Friends (Yes) | | |  |  |  |
|  | Wave 1 | 189 | 36.99 | 3.22 | 3.22 |
|  | Wave 2 | 214 | 47.03 | 13.83 | 3.60 |
|  | Wave 3 | 194 | 48.62 | 24.43 | 6.78 |
|  | Wave 4 | 180 | 48.00 | 28.98 | 7.41 |
|  | Wave 5 | 144 | 39.78 | 31.44 | 9.73 |
| Going for a Meal with Friends (Yes) | | |  |  |  |
|  | Wave 1 | 184 | 36.08 | 3.41 | 3.41 |
|  | Wave 2 | 233 | 51.21 | 13.83 | 3.60 |
|  | Wave 3 | 197 | 49.37 | 24.43 | 6.78 |
|  | Wave 4 | 174 | 46.40 | 28.98 | 7.41 |
|  | Wave 5 | 156 | 43.09 | 31.44 | 9.73 |
| Helping Friends with a Task (Yes) | | |  |  |  |
|  | Wave 1 | 105 | 20.55 | 3.22 | 3.22 |
|  | Wave 2 | 130 | 28.57 | 13.83 | 3.60 |
|  | Wave 3 | 111 | 27.82 | 24.43 | 6.78 |
|  | Wave 4 | 97 | 25.87 | 28.98 | 7.41 |
|  | Wave 5 | 101 | 27.90 | 31.44 | 9.73 |
| *Note*. Frequency estimates were based on non-imputed data. Range = possible score range. Full analytic sample N = 528. * N wave = 528, N wave 2 = 472, N wave 3 = 428, N wave 4 = 405, N wave 5 = 401. The placement of Psychological Wellbeing subscales (Mastery and Purpose in Life) within the surveys varied across the waves. | | | | | |
