# Supplementary material for: The value of social networks for men: concurrent and prospective associations with psychological wellbeing
Source: BMC Psychol. 2025 Feb 20;13:142. doi: 10.1186/s40359-025-02467-9 (PMC11843785; doi:10.1186/s40359-025-02467-9)
Supplement: Supplementary file 2 — Supplementary Material 2. [file 40359_2025_2467_MOESM2_ESM.docx]

| Table 2. Linear GEE Model of Social Network Variables Predicting Environmental Mastery across five waves (*n* = 528) | | | | | | | | | | | | |
| --- | --- | --- | --- | --- | --- | --- | --- | --- | --- | --- | --- | --- |
| Variable | Cross-sectional | | | | | | Longitudinal* | | | | | |
|  | Confounder unadjusted | | | Confounder adjusted | | | Confounder unadjusted | | | Confounder adjusted | | |
|  | *b* | 95% CI | *p* | *b* | 95% CI | *p* | *b* | 95% CI | *p* | *b* | 95% CI | *p* |
| **Level of Investment** |  |  |  |  |  |  |  |  |  |  | | |
| **Time Spent** | 0.13 | 0.08, 0.17 | **<.001** | 0.07 | 0.01, 0.16 | **<.001** | 0.05 | 0.01, 0.09 | **.018** | 0.06 | 0.02, 0.10 | **.005** |
| **Close Network Size** | 0.34 | 0.25, 0.43 | **<.001** | 0.32 | 0.23, 0.42 | **<.001** | 0.17 | 0.08, 0.26 | **<.001** | 0.16 | 0.07, 0.26 | **.001** |
| **Extended Network Size** | 0.13 | 0.08, 0.17 | **<.001** | 0.13 | 0.07, 0.18 | **<.001** | 0.06 | 0.02, 0.11 | **.009** | 0.07 | 0.02, 0.12 | **.003** |
| **Type of Investment** |  |  |  |  |  |  |  |  |  |  | | |
| **Physical Activity** | 1.43 | 0.85, 2.02 | **<.001** | 1.3 | 0.73, 1.90 | **<.001** | 0.26 | -0.24, 0.77 | .305 | 0.18 | -0.34, 0.70 | .504 |
| **Drink Alcohol** | 0.44 | -0.09, 0.97 | .106 | 0.41 | -0.12, 0.95 | .128 | 0.18 | -0.29, 0.65 | .459 | 0.13 | -0.34, 0.61 | 580 |
| **Go for a Meal** | 1.40 | 0.89, 1.90 | **<.001** | 1.3 | 0.80, 1.83 | **<.001** | 0.13 | -0.37, 0.62 | .617 | 0.06 | -0.47, 0.59 | .826 |
| **Help with a Task** | 0.73 | 0.20, 1.25 | **.007** | 0.73 | 0.20, 1.25 | **.007** | 0.14 | -0.46, 0.73 | .653 | 0.16 | -0.43, 0.74 | .604 |
| *Note.* All analyses adjusted for wave. Confounder adjusted analyses additionally adjusted for birthplace; education; income. Analyses are based on available case data. *In longitudinal analyses lagged predictors were assessed waves 1-4, one wave prior to outcomes and included additional adjustment for prior reports of environmental mastery. | | | | | | | | | | | | |

| Table 3. Linear GEE Model of Social Network Variables Predicting Purpose in Life across 5 Waves (*n* = 528) | | | | | | | | | | | | |
| --- | --- | --- | --- | --- | --- | --- | --- | --- | --- | --- | --- | --- |
| Variable | Cross-sectional | | | | | | Longitudinal* | | | | | |
|  | Confounder unadjusted | | | Confounder adjusted | | | Confounder unadjusted | | | Confounder adjusted | | |
|  | *b* | 95% CI | *p* | *b* | 95% CI | *p* | *b* | 95% CI | *p* | *b* | 95% CI | *p* |
| **Level of Investment** |  | | | | | | | | | | | |
| **Time Spent** | 0.06 | 0.02, 0.10 | **.007** | 0.07 | 0.03, 0.12 | **<.001** | 0.03 | 0, 0.07 | .13 | 0.04 | 0, 0.08 | **.051** |
| **Close Network Size** | 0.20 | 0.12, 0.28 | **<.001** | 0.20 | 0.11, 0.28 | **<.001** | 0.12 | 0.06, 0.18 | **<.001** | 0.11 | 0.03, 0.18 | **.007** |
| **Extended Network Size** | 0.04 | -0.01, 0.09 | .096 | 0.04 | -0.01, 0.09 | .119 | 0.04 | 0.01, 0.08 | **.009** | 0.05 | 0.02, 0.08 | **.002** |
| **Type of Investment** |  | | | | | | | | | | | |
| **Physical Activity** | 0.73 | 0.27, 1.19 | **.002** | 0.65 | 0.19, 1.10 | **.006** | 0.18 | -0.24, 0.60 | .416 | 0.21 | -0.20, 0.63 | .311 |
| **Drink Alcohol** | -0.07 | -0.53, 0.40 | .775 | -0.05 | -0.51, .40 | .816 | 0.27 | -0.11, 0.66 | .165 | 0.28 | -0.11, 0.70 | .156 |
| **Go for a Meal** | 0.91 | 0.47, 1.36 | **<.001** | 0.89 | 0.44, 1.34 | **<.001** | 0.18 | -0.25, 0.61 | .411 | 0.21 | -0.19, 0.61 | .296 |
| **Help with a Task** | 0.71 | 0.28, 1.15 | **.001** | 0.74 | 0.31, 1.16 | **.001** | 0.04 | -0.38, 0.46 | .851 | 0.02 | -0.40, 0.45 | .909 |
| *Note*. All analyses adjusted for wave. Confounder adjusted analyses additionally adjusted for birthplace; education; income. Analyses are based on available case data. *In longitudinal analyses lagged predictors were assessed waves 1-4, one wave prior to outcomes and included additional adjustment for prior reports of purpose in life. | | | | | | | | | | | | |
